# High-Resolution, Non-Invasive Imaging of Upper Vocal Tract Articulators Compatible with Human Brain Recordings

**DOI:** 10.1371/journal.pone.0151327

**Published:** 2016-03-28

**Authors:** Kristofer E. Bouchard, David F. Conant, Gopala K. Anumanchipalli, Benjamin Dichter, Kris S. Chaisanguanthum, Keith Johnson, Edward F. Chang

**Affiliations:** 1 Biological Systems and Engineering Division & Computational Research Division, Lawrence Berkeley National Laboratories (LBNL), Berkeley, California, United States of America; 2 Department of Neurological Surgery, University of California San Francisco (UCSF), San Francisco, California, United States of America; 3 Center for Integrative Neuroscience, UCSF, San Francisco, California, United States of America; 4 Department of Linguistics, University of California (UCB), Berkeley, California, United States of America; The University of Western Ontario, CANADA

## Abstract

A complete neurobiological understanding of speech motor control requires determination of the relationship between simultaneously recorded neural activity and the kinematics of the lips, jaw, tongue, and larynx. Many speech articulators are internal to the vocal tract, and therefore simultaneously tracking the kinematics of all articulators is nontrivial—especially in the context of human electrophysiology recordings. Here, we describe a noninvasive, multi-modal imaging system to monitor vocal tract kinematics, demonstrate this system in six speakers during production of nine American English vowels, and provide new analysis of such data. Classification and regression analysis revealed considerable variability in the articulator-to-acoustic relationship across speakers. Non-negative matrix factorization extracted basis sets capturing vocal tract shapes allowing for higher vowel classification accuracy than traditional methods. Statistical speech synthesis generated speech from vocal tract measurements, and we demonstrate perceptual identification. We demonstrate the capacity to predict lip kinematics from ventral sensorimotor cortical activity. These results demonstrate a multi-modal system to non-invasively monitor articulator kinematics during speech production, describe novel analytic methods for relating kinematic data to speech acoustics, and provide the first decoding of speech kinematics from electrocorticography. These advances will be critical for understanding the cortical basis of speech production and the creation of vocal prosthetics.

## Introduction

The ability to communicate through spoken language involves the generation of a wide array of sounds [1–3]. Speech sounds are produced by the coordinated movements of the speech articulators, namely the lips, jaw, tongue, and larynx [4]. Each articulator itself has many degrees of freedom resulting in a large number of vocal tract configurations. The precise shape of the vocal tract dictates the produced acoustics- however, at a coarse level, the same phoneme can be produced by many vocal tract configurations [5–9]. For example, normal production of the vowel /u/ involves raising the back of the tongue towards the soft palate while protruding/rounding the lips. Furthermore, the shape and size of individuals’ vocal tracts can vary significantly [10], and as a result there is not a general (i.e. cross-subject) mapping from vocal tract configuration and resulting acoustics that is valid across speakers [10,11]. Therefore, the precise shape of the vocal tract cannot be determined from observation of the acoustics alone. Furthermore, not all vocal tract movements have simultaneous acoustic consequences. For example, speakers will often begin moving their vocal tract into position before the acoustic onset of an utterance [12,13]. Thus, the timing of movements cannot be derived from the acoustics alone. This ambiguity in both position and timing of articulator movements makes studying the precise cortical control of speech production from acoustics measurements alone very difficult.

To study the neural basis of such a complex task requires monitoring cortical activity at high spatial and temporal resolution (on the order of tens of milliseconds) over large areas of sensorimotor cortex. To achieve the simultaneous high-resolution and broad coverage requirements in humans, intracranial recording technologies such as electrocorticography (ECoG) have become ideal methods for recording spatio-temporal neural signals [14–20]. Recently, our understanding of the cortical control of speech articulation has been greatly enriched by the utilization of electrocorticography (ECoG) in neurosurgical patients However, previous studies have only been able to examine speech motor control as it relates to the produced speech tokens, canonical descriptions of articulators, or measured acoustics, rather than the actual articulatory movements [14–20]. To date there have been no studies that relate neural activity in ventral sensorimotor cortex (vSMC) to simultaneously collected vocal tract movement data, primarily because of the difficulty of combining high-resolution vocal tract monitor with ECoG recordings at the bedside. The inability to directly relate to articulator kinematics is a serious impediment to the advancement of our understanding of the cortical control of speech.

In this study, our primary goal was to develop and validate a minimally invasive vocal tract imaging system. Additionally, we use novel, data-driven analytic approaches to better capture the shape of the articulators; synthesize perceptible speech from kinematic measurements; and combine our articulator tracking system with ECoG recordings to demonstrate continuous decoding of articulator movements. We collected data from six normal speakers during the production of isolated vowels (e.g. */ɑ/*, */i/*, */u/*, */ɝ/*) while simultaneously monitoring the lips, jaw, tongue, and larynx utilizing a video camera, ultrasound, and electroglottogram (EGG), respectively. We categorically related the measured kinematics to vowel identity and continuously mapped these measurements to the resulting acoustics, which revealed both shared as well as speaker specific patterns of vowel production. Application of unsupervised, non-negative matrix factorization (NMF) extracted bases that were often found to be associated with a particular vowel, and moreover allowed for a more accurate classification of vowels than traditional point-based parameterization of the articulators. Additionally, we synthesized auditory speech from the measured kinematic features and shows that these synthesize sounds are perceptually identifiable by humans. Finally, we demonstrated the feasibility of combining our noninvasive lip/jaw tracking system with ECoG recordings in a neurosurgical patient and demonstrate continuous decoding of lip-aperture using neural activity from ventral sensorimotor cortex. Together, our results suggest the methods described here could be used to synthesize perceptually identifiable speech from ECoG recordings.

## Materials and Methods

This study was approved by the UCSF Committee on Human Research. All participants gave their written informed consent before participating. The individual in this manuscript has given written informed consent (as outlined in PLOS consent form) to publish these case details.

### Task

Six speakers (5 males, 1 female) participated in this experiment. In order to mimic the conditions of a hospital bed, speakers sat at an incline with a laptop positioned at eye level ~0.25–0.5m away. To validate our articulatory monitoring system we had speakers produce vowels because they are well studied in the phonetics literature and much is known about their acoustics and articulatory bases [21–25]. Furthermore, the relationship between the shape of the vocal tract and the produced acoustics is the most direct for held vowels. Speakers were randomly presented with an audio recording (speaker KJ) of one of nine vowels (*ɑ/æ/ʌ/ɛ/ɝ/ɪ/i/ʊ/u*) in an hVd context (e.g. ‘hood’) and then in isolation [26]. Speakers repeated these tokens as they were presented following a brief (1 s) delay. For each speaker, between 30 and 50 repetitions of each vowel were collected. Only the isolated condition is examined here.

### Data Acquisition and Analysis

**Fig 1:**
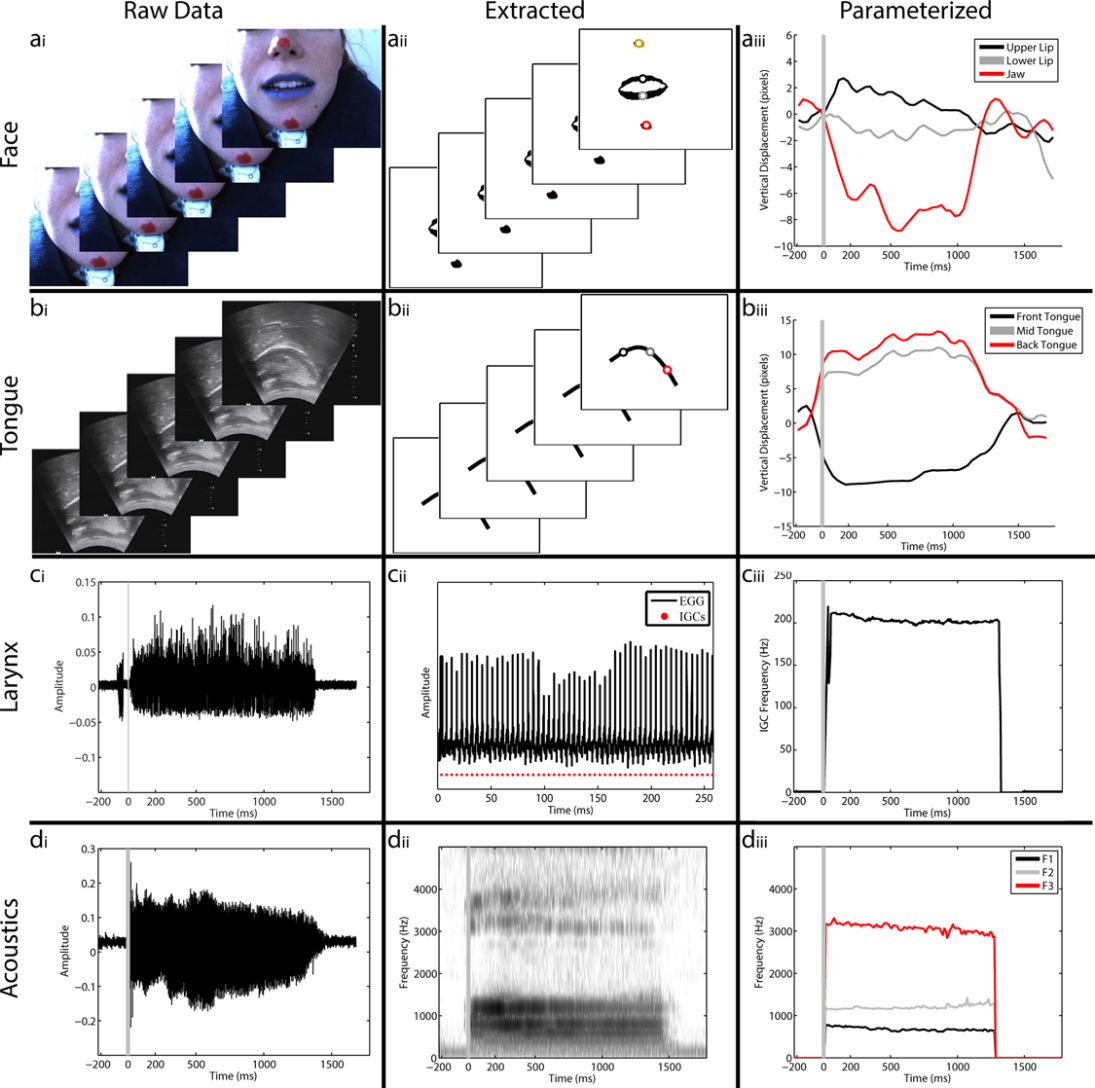
Data processing steps for facial, lingual, laryngeal, and acoustic data. **a)** The lips of the speaker were painted in blue and dots were painted in red on the nose and chin. A camera was then placed in front of the speaker’s face such that all painted regions were contained within the frame and the lips are approximately centered. Video was captured at 30 frames per second (fps) during speaking (i). Each frame of the video was thresholded based on hue value, resulting in a binary mask. Points were defined based upon the upper, lower, left and right extents of the lip mask and the centroids of the nose and jaw masks (ii). The X and Y position of these points was extracted as a time varying signal (iii). Grey lines mark the acoustic onset. **b)** The tongue was monitored using an ultrasound transducer held firmly under the speaker’s chin such that the tongue is centered in the frame of the ultrasound image. Video output of the ultrasound was captured at 30 fps (i). The tongue contour for each frame was extracted using EdgeTrak, resulting in an X and Y position of 100 evenly placed points along the tongue surface (ii). From these 100 points, three equidistant points were extracted, representing the front, middle, and back tongue regions which comprises our time varying signal (iii). **c)** Instances of glottal closure were measured using an electroglottograph placed with contacts on either side of the speaker’s larynx. The instances of glottal closure were tracked by changes in the impedance between the electrodes using the SIGMA algorithm [28]. **d)** Speech acoustics were recorded using a microphone placed in front of the subject’s mouth (though not blocking the video camera) and recorded at 22 kHz (Fig 1di). We measured the vowel formants, F_1_-F_4_, as a function of time for each utterance of a vowel using an inverse filter method. For the extraction of F_0_ (pitch), we used standard auto-correlation methods.

During the production of each sound, we simultaneously tracked the produced acoustics, as well as the movement of the vocal tract (lips, jaw, tongue, and larynx) employing three imaging methods. First, in order to capture the movement of the lips and jaw, the lips of the speaker were painted blue and red dots were painted on the nose and chin (Fig 1Ai) and a camera (FPS = 30) was placed in front of the speaker’s face such that all painted regions were contained within the frame and the lips are approximately centered [27]. In each frame of the video, lips and jaw position were determined using a hue threshold to extract the blue and red face regions, resulting in binary masks (Fig 1Aii). From the binary masks, we extracted the location of the jaw and the four corners of the mouth (upper/lower lip, left/right corners). The x and y position of these points were extracted as a time varying signal (Fig 1Aiii).

To image the tongue, an ultrasound transducer (Mindray M7 with C5-2s transducer) was held firmly under the speaker’s chin such that the tongue was centered in the frame (Fig 1Bi). Video for both the camera and the ultrasound was captured at 30 frames per second (fps). The tongue contour for each frame was extracted using EdgeTrak, which uses a deformable contour model and imposes constraints of smoothness and continuity in order to extract the tongue from noisy ultrasound images [29]. The output is an x and y position of 100 evenly placed points along the tongue surface (Fig 1Bii). Except where stated otherwise, our analyses parameterize tongue position as the vertical position of three equidistant points representing the front, middle, and back tongue regions (Fig 1Biii).

The larynx was monitored using an electroglottogram (EGG) (EG2-PCX, Glottal Enterprises). The subject wore a band around the neck, and the EGG measured the electrical impedance across the larynx with electrodes in the neckband on either side of the thyroid. The opening and closing of the glottis during voiced speech creates changes in the impedance (Fig 1Ci). The instants of glottal closure (IGCs) in the EGG signal were found using the SIGMA algorithm (Fig 1Cii) [28]. EGG recordings were collected on 3 of the 6 speakers.

Speech sounds were recorded using a Sennheiser microphone placed in front of the subject’s mouth (though not blocking the video camera) and recorded at 22 kHz (Fig 1Di). The recorded speech signal was transcribed off-line using Praat (http://www.fon.hum.uva.nl/praat/). We measured the vowel formants, F_1_-F_4_, as a function of time for each utterance of a vowel using an inverse filter method (Fig 1Diii). [30,31]. Briefly, the signal was inverse filtered with an initial estimate of F_2_ and then the dominant frequency in the filtered signal was used as an estimate of F_1_. The signal was then inverse filtered again, this time with an inverse of the estimate of F_1_, and the output was used to refine the estimate of F_2_. This procedure was repeated until convergence and was also used to find F_3_ and F_4_. The inverse filter method converges on very accurate estimates of the vowel formants, without making assumptions inherent in the more widely used linear predictive coding (LPC) method. For the extraction of F_0_ (pitch), we used standard auto-correlation methods. Instants of glottal closure (IGCs) in the acoustic signal were estimated from the acoustics using the DYPSA algorithm [32]. To adjust for differences in utterance duration, we used linear interpolation to temporally warp each trial for all extracted features (not the raw signals) such that it was equal to the median trial duration.

### Correlation Coefficient

We used the Pearson product-moment correlation coefficient (*R*) to quantify the linear relationship between two variables (x and y):

$$R(x,y)=\frac{cov(x,y)}{\sigma_{x}\sigma_{y}}$$ (1)

Where σ_x_ and σ_y_ are the sample standard deviations of x and y, respectively.

### Registration of kinematic data

To maximize the amount of data collected, subjects were recorded on multiple ‘blocks’, each lasting 10–15 minutes (as in the clinical setting). Different blocks could be collected on different days, and could be set-up by different experimenters. Furthermore, during a given block, there could be substantial movement of the subject, as well as the experimenter holding the ultrasound. All of these are potential sources of artifactual variability (noise) in the data. Therefore, a necessary pre-processing step is to ‘register’ all the data so as to minimize this noise. For each trial (across all recording sessions), the optimal affine transformation (shifts, rotations, and scaling) was found to maximize the overlap (minimize the difference) of each pre-vocalization image to the median of all pre-vocalization images. This transform was then applied to all subsequent time points. We found this optimal transform in two ways (each with their potential shortcomings): 1) grid-search over parameter ranges of entire binary images, 2) analytic calculation of transform for extracted features; both methods gave similar results. The details of these different approaches are described below.

The first approach was to calculate the optimal translation (**τ**) for a grid of rotations (**θ** = [-20^o^: 1: 20^o^]) and uniform scaling’s (**β** = [0.8: 0.01: 1.2]). For each image (**X**_i_), first we convolved the binary image with radial Gaussian (N(0,3)) for smoothing. For the face data, we then centered each image by assuming that the center of the mouth is in the same location. After this initial data processing, we calculated the reference image (X*) as the grand median across all the pre-vocalization images. We refined X* over three iterations of the following procedure. For each **X**_i_, we looped over both **θ** and **β** ranges, applied the transformations to the image, and performed 2D cross-correlations against X* to find the translation **τ**. For each image **X**_i_, the [**θ**, **β**, **τ]** triplet that gave the maximum cross-correlation values was taken as the optimal transformation:

$$\underset{\beta ,R,\tau }{\max}\;R(X^{*},X_{i})$$ (2)

This was applied to the data and then we re-calculated the reference image as the grand median of this transformed data, and re-ran the above procedure (using the non-transformed images). This was done to ensure the use of an optimal reference image, which is important for our reference based registration method. From this optimal reference image, the optimal [**θ**, **β**, **τ]** triplet was found as described above, and applied to the data. While slow, this method considers all the data in the image and so is not sensitive to feature extraction variability. Indeed, this method does not require any features to be extracted from the data, and so could be run with out defining ROI’s required for feature extraction. Furthermore, this method has lots of natural parallelization, both over parameters and trials, so compute time should decrease very well on cluster computing platforms.

The second approach for registering the data was to perform a so-called Procrustes analysis of the extracted landmarks in the images [33]. For this analysis, we used the x,y coordinates of the extracted tongue contours (100 points) and the 6 landmark points on the face (nose, jaw, upper/lower lip, left/right corners of lip). The Procrustes problem is to find the uniform scaling (**β**), rotation matrix (**θ**), and translation (**τ**) that minimizes the difference between a set of points (**X**_i_) and a reference set (X*):

$$\underset{\beta ,R,\tau }{\min}\;\frac{1}{2}{\left\|X^{*}-\beta X_{i}\theta +1\tau^{T}\right\|}_{F}^{2}$$ (3)

Where ||X||^2^_F_ = trace(**X**^T^**X**) denotes the squared Frobenius matrix norm.

Analogously to the grid-search procedure described above, we used the average shape across all pre-vocalization times as the reference set (X*) and we determined the Procrustes average of the pre-data data using an iterative procedure. First, we initialized X* to be the grand mean of the data points. Second, we solved the Procrustes problem (Eq 3) for each trial (**X**_i_) using the reference X*. Third, we applied the transform to each trial, and updated X* as the grand mean of the transformed data points. This procedure was repeated until numerical convergence of X* (here, taken to be **ΔX** < 10^−10^). With this optimized average shape calculated, we then solved and store the transformations from the Procrustes problem (Eq 3) for each trial, and applied to subsequent time points for that trial. This method has the advantage of being computationally fast, but for the face, depends on good extraction of features.

We examined the cross-utterance phoneme separability (Bouchard et al., 2013) of different vowels based on extracted parameter values before and after this registration. This quantifies the difference in the average distance between vowels and the average distance within a vowel, so larger values correspond to tighter distributions within a vowel and larger distances between vowels. We also used this metric to visually compare the time-courses of vowel identity structure in the acoustic and kinematic data. For a given feature set, phoneme separability (PS) is defined as the average difference of the median cross-cluster distances (*AC*) and median within-cluster distances (*WC*).

$$PS=\bar{AC-WC}$$ (4)

This measure expresses the average distance between vowels categories and the tightness of the category clusters.

### Classification

As described below, we used Naïve Bayes classifiers based on a latent dimensional representation (found by LDA) of acoustic, kinematic, and ‘shape’ features. These classifiers were trained and tested using a bootstrapped cross-validation procedure.

We applied Linear Discriminant Analysis (LDA) to the mean data from the central 1/5^th^ of time-points, using vowels as class identifiers. LDA is a supervised dimensionality reduction algorithm that finds the projection that maximizes the linear discriminability of the (user defined) clusters. LDA can be thought of as a discrete version of the general linear models used for continuous mapping (see below). Multiclass LDA was performed on the (central 1/5^th^ of) vowel acoustics, articulator features, and NMF representations by computing the matrix L* = **LS**^-1/2^, where **L** and **S** are the class centroids and common with-in class covariance matrices, respectively. Classes were the different vowels. We then took the singular-value decomposition of the covariance matrix of L*, and projected the data into the corresponding eigenspace (note that LDA necessarily results in a n-1 dimensional space). To ease comparisons across different speech representations, the first 3 latent dimensions (**L**^3^) were used for all feature sets (e.g. kinematic, acoustic, NMF).

We trained and tested Naïve Bayes classifiers to predict vowel identity based on different features of the produced vowels. The Naïve Bayes approach makes the simplifying assumption that each of the input features (in our case, projections of data into latent dimensions found by LDA) are conditionally independent, given the class identity (in our case, vowel identity). Under this assumption, the posterior probability of the class (V_k_) given the features (F_i_) is:

$$P(V_{k}|F_{i})=\frac{P(V_{k})}{{P(F}_{i})}\prod_{i}P(F_{i}|V_{k})$$ (5)

We used the *maximum a posterior* (MAP) as an estimator of class identity:

$$\hat{V}=\underset{k\in K}{\max}\;\frac{P(V_{k})}{{P(F}_{i})}\;\prod_{i}P(F_{i}|V_{k})$$ (6)

Here, we used a Naïve Bayes classifier as opposed to the posterior probabilities from the LDA because it gave slightly better performance.

### Bootstrapped cross-validation procedure

To train and test the Naïve Bayes classifiers, we used cross-validation on randomly selected (with replacement) subsets of the data. Specifically, within a 50 iteration bootstrap procedure, random 80% subsets of the data were used to train the classifiers, and model performance was tested on the 20% of data not used in (any part) of the training procedure. Performances are reported as the statistics (e.g. mean) across these bootstrap samples.

### Non-negative matrix Factorization

A common method for unsupervised learning of reduced basis sets is principal components analysis (PCA), which finds an orthogonal basis set that optimally captures the directions of highest variance in the data. However, a critique of PCA is that the bases often bear little resemblance to the data from which they were derived [34]. Although this may be of little consequence if quantitative performance is the primary interest (as is often the case in machine learning), when understanding the bases is important (as is often the case in science), this lack of resemblance to data can hinder interpretability [34]. Non-negative matrix factorization (NMF) has been used to extract ‘meaningful’ bases from data that consist of only positive values, such as images and movies (as in our data set)[34–36]. Additionally, NMF can be formally related to K-means clustering, and can result in clusters that are more robust than K-means [36]. NMF is a dimensionality reduction technique that extracts a predetermined number of bases ($B\in R^{mxk}$) and weights ($W\in R^{nxk}$) that linearly combine to reconstruct the non-negative data ($A\in R^{mxn}$), such that k < min(n,m) under the constraint that both the bases and weights are strictly non-negative:

$$A\approx BW^{T};\;B,W\geq 0$$ (7)

The solutions **B** and **W** are found by solving the constrained optimization problem:

$$\hat{B},\hat{W}=\underset{B,W}{\min}\;\frac{1}{2}{\left\|A-BW^{T}\right\|}_{F}^{2};\;s.t.\;B,W\geq 0$$ (8)

This matrix factorization has no closed form solution, and so is often found through numerical approximation (here, we used the Matlab ‘nnmf’ function). Additionally, NMF does not have a unique solution, and so good initialization is important. Therefore, we performed an initial search using the computationally less expensive (but less robust) multiplicative method with 20 random initializations and terminated the optimization procedure at 10 iterations or a numerical tolerance in the solution of 10^−10^. The result of this search that had minimal final reconstruction error was used as the initial conditions in a more exhaustive search using the non-negative alternating least-squares approach. Here, the procedure terminated after 100 iterations or a numerical tolerance of 10^−16^.

We applied this procedure separately to the (registered) face and tongue images from the central 1/5^th^ of each utterance. We applied NMF to the data for faces and tongues separately because the NMF objective function (Eq 8) finds solutions that directly minimize the pixel level reconstruction of the raw data. Therefore, because the number of pixels associated with the tongues and lip images are very different, a combined analysis would result in differential weighting of these articulators in the extraction of the basis. Indeed, the number of non-zero pixels in the lip data is much greater than the tongue data, indicating that lips would be given a much larger importance in the NMF reconstruction, which is undesirable.

### Linear Mapping

To understand the continuous relationship between articulator position and resulting acoustics, we utilized general linear models. For each trial, the average value over the middle fifth of the vowel was calculated for each articulatory and acoustic feature. These averages were then z-scored across trials to remove differences in scaling between recording modalities. We then used the Boostrapped Adaptive Threshold Selection (BoATS) algorithm to estimate regularized linear models using an 80-10-10 cross-validation procedure to derive model weights from training data (80% of data), determine an optimal regularization parameter (10% of data) and calculate model performance on test data (10% of data) [14,37]. Briefly, first, to derive null distributions of weights (β*^rnd^) and model performance (R^2^_rnd_), we randomly permuted (200 times) each input feature independently relative to the output feature on a trial-by-trial basis. Second, within a 200 iteration bootstrap procedure, random 80% subsets of the data were used to derive linear weights for the models using the equation:

y=βX (9)

Where y is the predicted feature, X is the set of predictor features, and β are the weights that describe the linear relationship. From this, we arrived at an estimate of weights (β*^obs^) for each input feature predicting and output feature. We then reduced the dimensionality of the input features (X) by comparing the model weights between the observed and randomized data sets to identify input features with weights that were different between the two conditions. Specifically, features (X_j_) were retained if the weight magnitude (|β_j_*^obs^|) was greater than the mean plus a threshold multiple (regularization parameter) of the standard deviation of the distribution of weight magnitudes derived from the randomization procedure (|β_j_*^rnd^|). Finally, we re-trained models on the training data based only on this reduced set of cortical features to arrive at optimal weights (β*^reg^) and determined decoding performance (R^2^_reg_) on test data (10%) not used in training. The choice of threshold was chosen to optimize the predictive performance on the regularization data (10%). The model performance was taken as the mean of R^2^_reg_ values across bootstrap test samples. This quantifies the expected value of predictive performance across randomly selected training and test samples.

### Speech Synthesis

Statistical parametric approaches are the dominant approach for speech synthesis in recent years for their flexibility in mapping arbitrary feature descriptions of speech and language to intelligible speech [38]. In traditional speech synthesis, text or sequences of phonemes are input, which are then analyzed to get relevant linguistic and contextual information into building a supervised model that optimizes the prediction of speech given its context. Since this approach assumes noiseless inputs of linguistic categories, it is not usable, as it is, for the current task. The contextual information in this study is continuous and articulatory, and noisy. This requires building a speech synthesizer that can work on such inputs to optimally predict speech. Clustering and Regression Trees (CART) [39] is a widely used model in statistical speech synthesis for mapping contextual feature representations into a synthesizable feature representation of speech. Speech parameters were extracted for each trial of vocalization from each subject. This comprises joint vectors of Fundamental Frequency (F_0_), Mel-Cepstral Coefficients, excitation strengths and voicing. This description is sufficient to resynthesize perceptually lossless speech [40]. In the current setting of estimating these representations from articulatory features, the context comprises continuous-valued questions about the spatial co-ordinates of various tracked features in the vocal tract (e.g lip-width > 3.8 units?). Based on the configuration of vocal tract considered, the articulatory streams include points on the tongue or lips or a combination of both to model the produced acoustics. These articulatory feature streams were resampled at the same frequency of the speech, so as to create aligned vectors for training and so that the synthesized acoustics were at the same sampling rate as the produced acoustics for perceptual comparisons.

The CART model itself is a decision tree that hierarchically clusters data in subsets that are optimally described by categorical or continuous valued questions about given aspects of the training data. Questions about the appropriate articulatory features were greedily chosen in CART training to best reduce the variance in the data due to the split. The variance reduction I_v_(N) for a node N, that split the acoustic data S into subsets S_t_ and S_f_ is given by

$$I_{v}(N)=\frac{1}{\left|S\right|}\sum_{i\in S}\sum_{j\in S}\frac{1}{2}{\left(x_{i}-x_{j}\right)}^{2}-\left(\frac{1}{\left|S_{t}\right|}\sum_{i\in S_{t}}\sum_{j\in S_{t}}\frac{1}{2}{\left(x_{i}-x_{j}\right)}^{2}+\frac{1}{\left|S_{f}\right|}\sum_{i\in S_{f}}\sum_{j\in S_{f}}\frac{1}{2}{\left(x_{i}-x_{j}\right)}^{2}\right)$$ (10)

The decision tree was recursively grown until a criterion is met, like the minimum number of data points within a cluster. A stop value of 50 was used, as the minimum number of data points within a subcluster. The mean and variance statistics of the subset of data points within the final clusters were stored at the leaf nodes of the trees. At runtime for synthesizing speech from given articulatory trajectories, the CART trees were traversed and the mean speech vectors at the leaf nodes were sequentially picked at the frame rate of the articulatory data and synthesized.

We report the performance of the model both objectively and subjectively. For objective quantification, we used the Mel-Cepstral Distortion (MCD) [41], which is a normalized sum Euclidian distance between a sequence of synthesized cepstral features and those of the corresponding reference acoustic stimuli. MCD for a reference 24-dimensional mel-cepstral vector and an estimate is given by

$$MCD=\frac{10}{ln(10)}\sqrt{\sum_{0<d<25}{\left({mc}_{d}^{\left(y\right)}-{mc}_{d}^{\left(\hat{y}\right)}\right)}^{2}}$$ (11)

For subjective evaluation, we used human subjective evaluation via the Amazon Mechanical Turk.

### Mechanical Turk for Subjective Assessment of Synthesis

Perceptual listening tests were conducted on the Amazon Mechanical Turk. The Mechanical Turk is a crowdsourcing portal where paid online volunteers perform tasks like annotations, perceptual judgments etc., called HITs (Human Intelligence Tasks). It is possible to constrain the task to be assigned to volunteers from a geographical region or those with a desired skill set or HIT success rate. To evaluate the speech synthesis outputs of different articulatory representations, a held out set of articulatory trajectories is synthesized and HITs are created such that qualified Turkers judge each synthesized audio stimulus. The task itself is vowel identification based on the audio of each stimulus. In this forced choice identification task, for each audio stimulus, Turkers were asked to choose one among nine vowels that best identifies the vowel as they perceived it. Illustrative examples of each vowel (e.g., */æ/* as in /*CAT/*) were also provided to help those without formal phonetic knowledge. While quality control is hard, some metrics like the HIT response time can be thresholded to weed out spammers among the volunteers. Unless reported otherwise, all listening tests were conducted with no restrictions on the location of the Turker. A HIT success rate of 80% was used to select only the genuine Turkers. HITs were randomly created and assigned such that each stimulus was identified by at least 10 Turkers. A HIT response time threshold of 30 seconds was used to filter out spurious Turkers.

### ECoG Subjects and Experimental Task

One native English speaking human participant underwent chronic implantation of a high-density, subdural electrocortigraphic (ECoG) array. Our recordings were from the language dominant hemisphere (as determined with the Wada carotid intraarterial amybarbital injection), which was the right hemisphere in this patient. Participants gave their written informed consent before the day of surgery. The participant read aloud a set of words and pseudo-words (e.g. ‘Leakst Skoot’**)** [27].

### Anatomical location of vSMC

We focused our analysis on the ventral (“speech”) portion of the sensory-motor cortex (vSMC). vSMC is anatomically defined as the ventral portions of the pre-central and post-central gyri, as well as the gyral formation at the ventral termination of the central sulcus, known as the sub-central gyrus. Visual examination of co-registered CT and MR scans indicate that the ECoG grid in the patient covered the spatial extent of vSMC [14,15].

### ECoG Data Acquisition and Signal Processing

Cortical surface field potentials were recorded with ECoG arrays and a multi-channel amplifier optically connected to a digital signal processor (Tucker-Davis Technologies, Alachua, FL). The spoken syllables were recorded with a microphone, digitally amplified, and recorded in-line with the ECoG data. ECoG signals were acquired at 3052 Hz. The microphone audio signal was acquired at 22kHz.

The time series from each channel was visually and quantitatively inspected for artifacts or excessive noise (typically 60 Hz line noise). Artifactual recordings were excluded from analysis, and the raw recorded ECoG signal of the remaining channels were then common average referenced. For each channel, the time-varying analytic amplitude was extracted from eight bandpass filters (Gaussian filters, logarithmically increasing center frequencies (70–150 Hz) and semi-logarithmically increasing band-widths) with the Hilbert transform. The high-gamma (High-γ) power was then calculated by averaging the analytic amplitude across these eight bands, and then this signal was down-sampled to 200 Hz. High-γ power was z-scored relative to the mean and standard deviation of baseline (i.e. subject at rest in silence) data for each channel. Throughout, when we speak of High-γ power, we refer to this z-scored measure, denoted below as Hγ.

Principal components analysis (PCA) was performed on the set of all vSMC electrodes for dimensionality reduction and orthogonalization. This also ensures that the matrices in the calculation of least mean squared error estimators (from regressions below) were well scaled. For each electrode (e_j_ of which there are n, n = 59) and time point (t, of which there are m), we calculated the high-gamma power. The Hγ(e,t) were used as entries in the n x m data matrix **D**, with rows corresponding to channels (of which there are n) and columns corresponding to the number of time points (of which there are m). Each electrode’s activity was z-scored across time to normalize neural variability across electrodes. PCA was performed on the n x n covariance matrix **Z** derived from **D**. The singular-value decomposition of **Z** was used to find the eigenvector matrix **M** and associated eigenvalues **λ**. The PCs derived in this way serve as a spatial filter of the electrodes, with each electrode e_j_ receiving a weighting in PC_i_ equal to m_ij_, the i-j^th^ element of **M**, the matrix of eigenvectors. For each point in time, we projected the vector Hγ(e,t) of high-gamma activity across electrodes into the leading 40 eigenvectors (**M**^40^):

$$\Psi (t)=M^{40}•H\gamma (e,t)$$ (12)

We used 40 PCs, which accounted for ~90% of the variance.

### Kinematic Feature Decoding Model

The Ψ(t) (Eq 12) served as the basis for training and testing optimal linear predictors of lip aperture (A(t)) over time using BoATS algorithm described above. We used a simple linear model to predict the lip aperture from Ψ(t-τ):

$$\hat{A}(t)=\beta •\Psi (t-\tau )+\beta_{0}$$ (13)

Where $\hat{A}(t)$ is the best linear estimate of A(t) based on the cortical features. The vector of weights β that minimized the mean squared error between $\hat{A}(t)$ and A(t) was found through multi-linear regression and cross-validation with regularization (see above). Based on our previous work [14], we used **τ** = 100ms.

### Statistical Testing

Results of statistical tests were deemed significant if the probability of incorrectly rejecting the null-hypothesis was less than or equal to 0.05. We used paired Wilcoxon sign-rank tests (WSRT) for all statistical testing.

## Results

We describe methods for acquisition and analysis of high-resolution kinematic data from the diverse set of vocal tract articulators that is compatible with human electrophysiology. For the initial characterization and validation of these methods we focused on data collected from speakers during the production of American English vowels, as these are a well studied and understood subset of speech sounds that engage the articulators monitored here. Specifically, we performed a variety of analyses to validate our methodology by comparing to previous results across a variety of domains, and propose new methods for measuring, parameterizing, and characterizing vocal tract movements. First, we describe techniques for reducing artifacts from recorded articulator videos, allowing us to combine data across different recording sessions. Next, we show the measured acoustics and articulator position time courses, and quantify the extent to which acoustic and kinematic features can discriminate vowel category, both of which are in good agreement with classical studies of vowel production. In line with the categorical conceptualization of speech, we describe a data-driven approach to extract vocal tract shape using non-negative matrix factorization (NMF). This method discovers ‘shapes’ that allow for more accurate classification of vowels than *a priori* defined parametric descriptions of the articulator positions. We then transition from categorical to continuous mappings between articulators and acoustics. Using the measured articulator positions, we assessed how articulatory features and acoustics linearly map to one another. Next, we synthesized speech from articulator positions and demonstrate that the processed articulatory trajectories retain sufficient signal to synthesize audio that can be perceived as the intended vowel. Finally, to illustrate the potential of combining articulatory tracking with brain recordings, we demonstrate robust decoding of a speech articulatory movement using multi-linear methods.

Our goal was to develop an articulatory tracking system compatible with electrocorticgraphy (ECoG) recordings at the bedside. This imposes several strong constraints on our experimental protocol. In particular, because our ECoG recordings are taken from neurosurgical patients, it is not possible to secure any apparatus to the patients’ head. Additionally, only a limited amount of data can be collected in a given recording session, and so data are often taken on multiple recording sessions. Finally, the recording equipment must be as electrically quiet as possible so as to not interfere with the electrical recordings from the brain. Thus, our recordings in non-clinical speakers were subject to the same experimental constraints and multi-session recordings. Our approach combined the simultaneous use of ultrasonography to track the tongue, videography to monitor the mouth and jaw, and electroglotiography to measure the larynx. The raw data from this system and initial extraction of vocal tract articulators and parametric tracking is displayed in Fig 1. We collected data from six American English speakers (5 male, 1 female) during the production of hVd (e.g. “hood”) words and sustained production of the corresponding vowels. The results presented here are focused on the vowel hold segment of the task, which included 1813 vocalizations (N = 292, 290, 197, 270, 391, 373 for the six speakers).

### Utterance-to-utterance registration of vocal tract data

**Fig 2:**
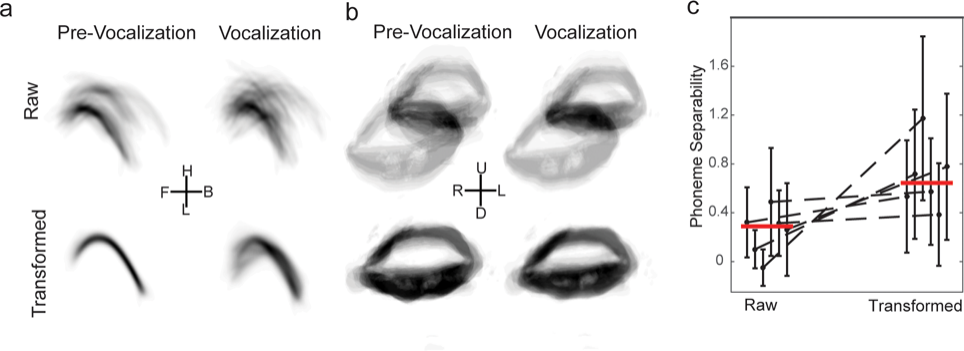
Utterance-to-utterance registration of vocal tract data. **a)** All tongue data from one subject. Left, top: overlay of raw tongue data during the pre-vocalization period for all trials; left, bottom: overlay of the same data after applying the optimal transformation. Right, top: overlay of raw tongue data during vocalization for all trials; right, bottom: overlay of the same data after applying the transformation from the pre-vocalization time. **b)** All lip data from one subject. Left, top: overlay of raw lip data during the pre-vocalization period for all trials; left, bottom: overlay of the same data after applying the optimal transformation. Right, top: overlay of raw lip data during vocalization for all trials; right, bottom: overlay of the same data after applying the transformation from the pre-vocalization time. **c)** Quantification of efficacy of applying transform from pre-vocalization data to vocalization times: enhanced separability. We calculated the separability between vowels based on articulatory features during vocalization before (raw) and after (transformed) applying the transformation optimized for the pre-vocalization time. The transformed data consistently had increased separability. Black points: mean ± s.d. across vowel comparisons for a subject, red line: median across subjects.

For both the ultrasound and videography recordings, a major source of artifactual variability introduced by our constraints was inconsistency in the position of the sensors (ultrasound transducer and camera), resulting in translations, rotations, and scaling differences in the plane of recordings. For example, the images shown in the top row of Fig 2A and 2B display the mean tongue and lip shapes extracted from the raw data at pre-vocalization times, as well as during the center (central 1/5^th^) of the vocalized vowels (N = 292 for all plots). In all plots, there are clear translations, rotations, and scaling differences between frames. (e.g. the translation and scaling of the mouth). These experimental artifacts are clearly a serious impediment to analyzing the data.

To correct for these experimental aberrations, we registered the images based on pre-vocalization frames with three simplifying assumptions: (1) the vocal tract is assumed to be the same across all vocalizations during pre-vocalization, (2) the position of the sensors is stable on the time-scale of a single vocalization, and (3) transformations are assumed to be affine (rotation, translation, and scaling). Ultrasound and videography from each trial were registered by first finding the transformations (translation, rotation, and scaling) that maximized the overlap of the pre-vocalization data (on an utterance-to-utterance bases), and then applying these transformations to subsequent time points. The details of this procedure are described in the Methods. Briefly, for each trial (across all recording sessions), the optimal affine transformation (translation, rotation, and scaling) was found to maximize the overlap of the pre-vocalization images to the median image (after an initial centering operation). This transform was then applied to all subsequent time points. We found this optimal transform in two ways (each with their potential shortcomings): 1) brute force search of binary images, 2) analytic calculation of affine transform for extracted features (i.e. ‘Procrustes Analysis’); both methods gave similar results.

We found that image registration removed much of the obviously artifactual variability in the images. The images shown in the bottom row of Fig 2A and 2B display the mean tongue and lip shapes after registration for pre-vocalization times, as well as during the center of the vocalized vowels. For the pre-vocalization data, the mean of the transformed images is clearly less variable then the mean of the unregistered extracted images for both the tongue (Fig 2A, left column) and the lips (Fig 2B, right column). For example, the large translation and scaling of the mouth have largely been removed. This validates that our procedure is working as expected. Importantly, applying the transformation optimized on pre-vocalization data to data during vocalization times greatly cleaned up these images as well (Fig 2A and 2B, right columns).

We checked that the transformations derived from the pre-vocalization data were removing artifactual variability while preserving signal useful for discriminating the different vowels. For this, we extracted articulatory features (e.g. lip aperture, front tongue height) from the central 1/5^th^ of each utterance. Based on these features, we calculated the separability between the different vowels, which measures the distance between the data for different vowels relative to the tightness of the data for the same vowel. In Fig 2C, we plot the separability for each subject before (raw) and after (transformed) applying the optimal transformation from the pre-vocalization data (black: mean ± s.d. for individual subjects; red lines, median across subjects). For each subject, the average separability of the vowels was enhanced by the application of the transformation. Importantly, the subjects with the greatest enhancement were those that had the worst separability before the transformation, indicating that the degree of enhancement scales with the amount of artifact present. Together, these analyses demonstrate that our method of data registration removes artifactual variability due to data acquisition and enhances the signal useful for differentiating the vowels. This allows us to combine data acquired across different recording sessions.

### Articulatory and Acoustic Feature Time-courses and Classification

To examine how the articulatory and acoustic measures change over the course of vowel production, we produced time-courses for each feature. This visualization allows for initial validation that our articulator monitoring system is producing meaningful measurements. For each trial, we extracted acoustic features (F_0_-F_4_) and articulatory features (front, mid, and back tongue, lip aperture and lip width) over the time-course of the vowel utterance. To eliminate differences in scale between features, we first z-scored each feature across all trials. For each speech feature, the average and variance was calculated across trials of the same vowel.

**Fig 3:**
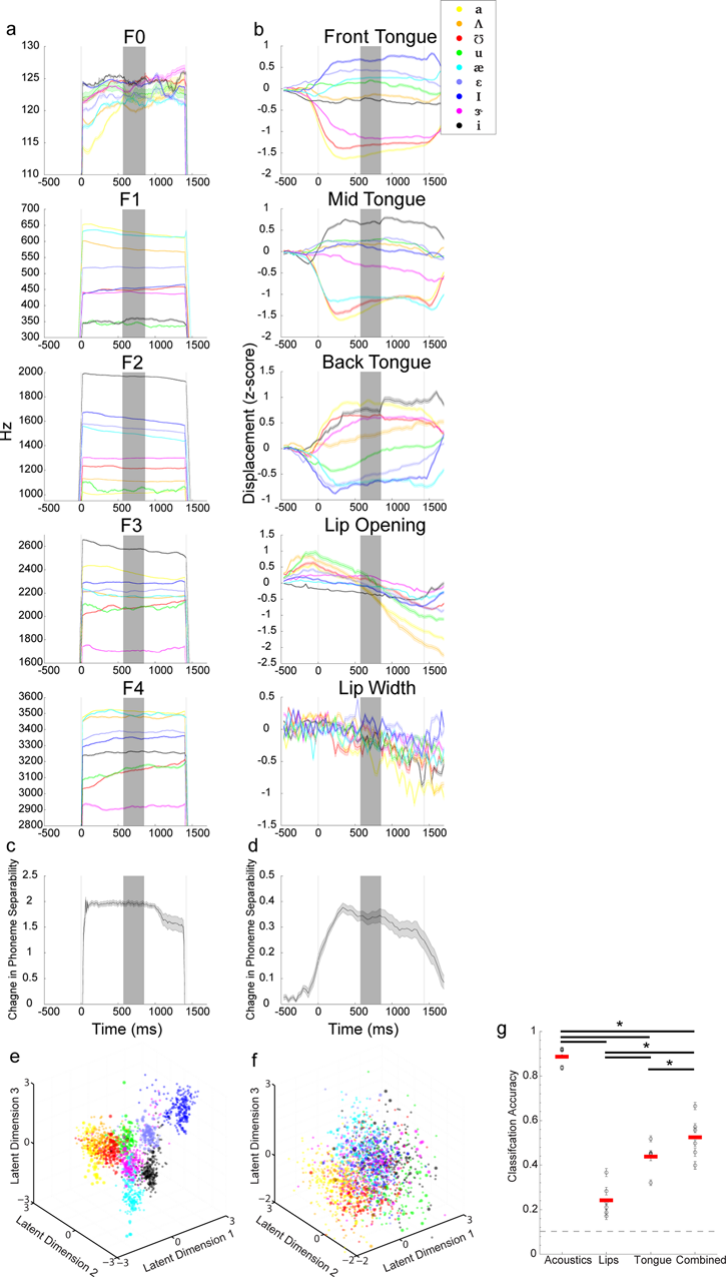
Articulatory and Acoustic Feature Time-courses and Classification. **a-b)** Average time course of formant values(a) and articulator position (b) for each of the 9 vowels examined. Traces shown are for a single subject (speaker 1). Each trial was warped using linear interpolation so that all trials were of equal length. Grey lines mark the acoustic onset and offset. Error bars denote standard error. Shaded region marks the time window used for LDA and classification analyses in (e-g). **c-d)** Change in cluster separability across the trial for acoustic (c) and articulatory (d) features. Error bars denote standard error. **e-f)** LDA projections of formant values (e) and articulator position (f) drawn from the middle fifth of each trial across all speakers. All values are z-scored across all trials. Each dot marks the values for a single trial. Color denotes vowel spoken during the trial. Larger dots mark trials from a single speaker (same as in Fig 4I). **g)** Classification performance resulting from running a 50x cross-validated naïve Bayes classifier on the mid-vowel acoustic and kinematic measurements. Each dot denotes an individual speaker with error bars denoting standard error across the cross-validations. Red line marks the median performance across speakers. Horizontal lines denote statistical significance (P < 0.05, WSRT, N = 6).

The acoustic and articulatory features for speaker 1 are plotted in Fig 3A and 3B. Each color shows the average trace for a different vowel, and error bars show standard error. The grey region marks the central 1/5^th^ of the vocalizations. For the acoustic features, F_1_ through F_4_ all exhibit considerable separation between vowels during steady state production, and the relative magnitudes between vowels are consistent with previous literature on vowel acoustics [2,24,26]. As an example, the vowel */i/* (black) has the highest F_2_, but very low F_1_. F_0_ (pitch) shows little separability between vowels, but does show a consistent pitch lowering for which is consistent with previous literature on intrinsic pitch [42]. For the articulatory features, tongue height and lip aperture also demonstrate clear vowel category structure, while lip width did not vary consistently between vowels. The lack of category structure in lip width measurements may partially reflect the fact that movements in lip width were small relative to lip opening and tongue movements. Interestingly, while tongue height measures all reach a steady state during production of the vowel, lip aperture continuously changes during the trial, reaching maximal opening around onset, and beginning to close during the production of the vowel. The positions of the speech articulators during vowel production are in line with previous descriptions [4,23,25,43,44]. For example, production of the vowel */ɑ/* resulted in lowering of the front and mid tongue points, and raising the back tongue, all consistent with the description of */ɑ/* being a ‘low-front’ vowel. The timing of movement onset is also similar to previous descriptions: most articulatory movement started shortly before acoustic onset, reached steady state shortly thereafter, and remained in position well past acoustic offset (i.e. end of phonation) [43]. Additionally, lip movements tend to precede tongue movements.

To quantify the dynamics of vowel category structure for both acoustic and kinematic features, we first performed linear discriminants analysis (LDA) on all the acoustic and kinematic features (see Methods). LDA is a dimensionality reduction method that finds the lower-dimensional manifold that allows best linear discriminability of the (pre-defined) categories (here, the vowels). We projected the acoustic and kinematic features into the first three dimensions of the LDA space, and calculated the phoneme separability at each time point. This allows comparison between acoustic and kinematic features in the same number of dimensions in an orthogonalized space (so Euclidean distance is valid). The phoneme separability was calculated for each time point for each subject, then averaged across subjects. In Fig 3C and 3D, we present the average separability for the acoustic (Fig 3C) and kinematic features (Fig 3D)(mean ± s.e.m. across subjects). For acoustic features (Fig 3C), phoneme separability between vowels exhibits steep onsets and offsets and remains steady during vocalization, which is expected given that there are no measureable acoustics outside of the vocalized region (by definition). For kinematic features (Fig 3D), phoneme separability is lower in magnitude and has a more gradual time course, rising before acoustic onset, peaking shortly after, and falling slowly after offset. Together, these time courses suggest that our method produces articulatory and acoustic measurements with reasonable timing and magnitude for each of the vowels measured. However, the difference between acoustic and kinematic time courses is an important issue to consider for understanding the cortical control of speech production. Specifically, there are clear movements of the articulators with no simultaneous acoustic consequences, emphasizing the importance of explicitly measuring articulator kinematics.

As the identity of a vowel is defined not by a single feature, but by the relationships amongst multiple features, we next visualized how the vowels clustered in multi-dimensional acoustic and kinematic spaces. We took the average feature value during the steady state portion of each vocalization (central 1/5th) for each articulatory and acoustic feature and labeled each trial according to the vowel spoken. In the acoustic space (Fig 3E), the different vowels shows very little overlap. In the kinematic space (Fig 3F) there are distinct regions for each vowel, but there is large overlap between vowels. The difference in overlap between kinematics and acoustics may partially be due to a larger degree of noise in the kinematic recordings. To quantitatively identify the features that best discriminate between vowels, we determined the contribution of each acoustic and articulatory feature to each of latent dimensions in the LDA space (S1 Fig). On average, the acoustic LDs primary contributions were from F_2_ for LD1, F_1_ for LD2, and F_3_ for LD3. The first two articulatory LDs are dominated by tongue height, while the third is predominantly lip opening (S1 Fig).

Finally, to quantify the extent to which acoustic and kinematic features can discriminate vowel category, we used a naïve Bayes classifier (see Methods) to predict vowel identity from the first 3 LDs derived from vowel acoustics, lip features alone, tongue features alone, and all kinematics (Fig 3G: black: mean and standard error for individual subjects; red lines: median across speakers). Acoustics are the best predictor of vowel category, with on average 88% correct classification, and classification based on the lips alone (24%), tongue alone (43%), and all kinematic features combined (52%), all performed significantly better than chance (11%) (*: P < 0.05, WSRT, N = 6). Importantly, performance of all kinematic features is significantly higher than either lip or tongue features alone (*: P < 0.05, WSRT, N = 6) demonstrating that there is non-redundant information between the lips and tongue. All of these findings are consistent with classic descriptions of the articulatory and acoustic bases of vowel, and provide further validation of our recording system and registration methods [24,26,43,45].

### Unsupervised extraction of vocal tract shape with non-negative matrix factorization improves vowel classification

The modest classification of vowel identity based on articulator kinematics could be due to a number of causes. For example, it is likely that, even with image registration, the measurement noise in our articulatory imaging system and extraction procedures is larger than that of the collected acoustics and formant extraction procedures. Alternatively, the transformation from articulator configurations to acoustics could be highly non-linear, or very small differences in the vocal tract shape could lead to large differences in the acoustic output. However, the poor performance could also reflect the parameterization we chose to describe the articulators. Although well motivated by the literature, this parameterization was not entirely data driven (i.e. it was determined by the experimenters, not derived from the data *de novo*), and does not capture the richness of the full articulator shapes.

**Fig 4:**
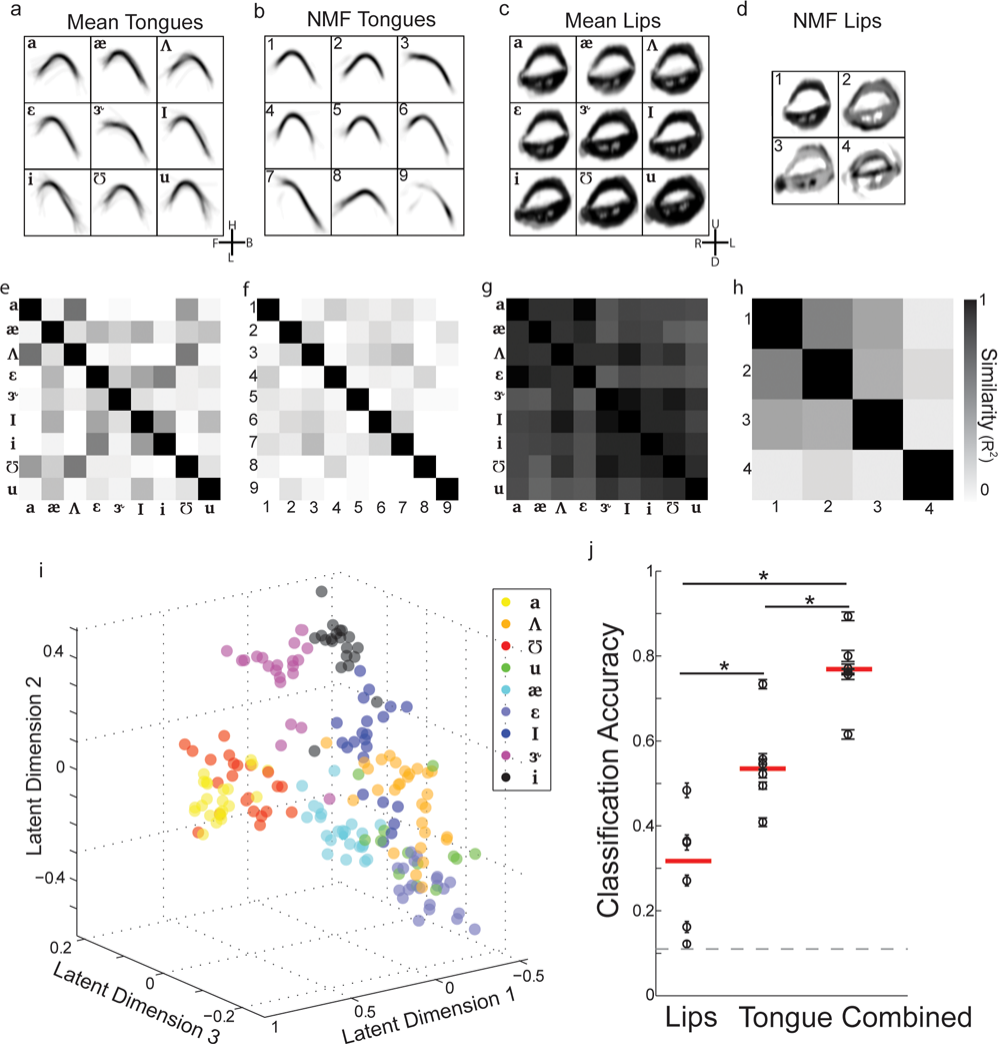
Unsupervised extraction of vocal tract shape with non-negative matrix factorization improves vowel classification. **a)** Mean tongue shape for each vowel from one subject. **b)** Non-negative matrix bases blindly extracted from the tongue data for all vowels from one subject. **c)** Mean lip shape for each vowel from one subject. **d)** Non-negative matrix bases blindly extracted from the lip data for all vowels from one subject. **e-h)** Similarity (R^2^) between mean tongue shapes (**e**), tongue non-negative matrix components (**f**), mean lip shapes (**g**), and lip non-negative matrix components (**h**). **i)** Scatter plot of all vowels in the first 3 linear discriminant dimensions for one subject (same as Fig 3A–3D). **j)** Cross-validated classification accuracy of vowels from vocal tract shapes across all subjects. Naïve Bayes classifiers were trained to predict vowel identity based on NMF reconstruction weights for the lips and tongue individually, as well as from both lips and tongue. The combined model out-performs the individual models. Furthermore, the classification accuracy is enhanced relative to using the pre-defined articulatory features.

One reasonable choice of basis images that describes the vocal tract would be the mean image associated with each vowel. For example, in Fig 4A and 4C, we plot the mean tongue and lip images (center 1/5^th^ of each vocalization) associated with each of the nine vowels in our data set from one speaker. These ‘bases’ clearly reveal that /*ɑ*/ is produced by a low-back tongue shape with an open lip configuration, /*i*/ is produced by a high-front tongue shape and a more narrow lip configuration, and **/***u*/ is produced by a high-back tongue shape and a narrow lip configuration. This ‘basis set’ has the advantage (by definition) that each individual basis can be readily associated with a given vowel, making them easily interpretable. However, from a mathematical perspective, using the mean tongue and lip images as bases have several undesirable properties: (1) they are supervised, requiring the vowel labels to be known beforehand, (2) they reflect the full variability of the data set, and thus can be sensitive to individual trials (e.g. light gray traces in /i/), (3) many of the average images are quite similar, and therefore it is unlikely that these images correspond to a parsimonious description of the data. This last point can be formalized by simply measuring the coefficient of determination (R^2^) between each image. The similarity matrices at the bottom of the Fig 4A plot the R^2^ values of all pair-wise comparisons of images. Several of the tongue images (e.g. Fig 4A bottom: /*ɑ*/ vs. /*ʌ*/ and /*i*/ and /*ɪ*/), and most of the lip images (Fig 4C bottom) have high-degrees of similarity.

We therefore used unsupervised learning methods to extract structure from the images that capture the entire shape of the tongue and lips with a reduced number of bases. A common method for unsupervised learning of reduced basis sets is principal components analysis (PCA), which finds an orthogonal basis set that optimally captures the directions of highest variance in the data. However, a critique of PCA is that the bases often bear little resemblance to the data from which they were derived [34]. Although this may be of little consequence if quantitative performance is the primary interest (as is often the case in machine learning), when understanding the bases is important (as is often the case in science), this lack of resemblance to data can hinder interpretability. Non-negative matrix factorization (NMF) has been used to extract ‘meaningful’ bases from data that consist of only positive values, such as images and movies (as in our data set) [34]. NMF is a dimensionality reduction technique that extracts a predetermined number of bases (B) and weights (W) that linearly combine to reconstruct the data, under the constraint that both the bases and weights are strictly non-negative [34].

As our study primarily focuses on examining the steady-state configurations of the vocal tract during the production of vowels, we applied NMF to the lip and tongue images extracted from the center of the vowel (see Methods). The plots in the top of Fig 4B display the leading nine NMF bases derived for the tongue data, while Fig 4D displays the leading four NMF bases derived from the lip data. Focusing first on the tongue, we found that nine NMF bases could accurately and parsimoniously reconstruct the single utterance images (reconstruction error plateaued at ~nine NMFs). Furthermore, NMF extracted many bases shapes that could readily be associated with a particular vowel. For example, basis 1 in Fig 4B is very similar to the mean image for /*æ*/, while basis 3 resembles the mean image for /*ɝ*/. We note that, although this is an intuitive solution for the algorithm, it is not guaranteed mathematically. Additionally, not only were many of these bases interpretable, but they also appeared to contain less ‘contamination’ from single trials than mean images. Finally, the different NMF bases were, generally speaking, less similar to each other than the mean images, which is quantified for this subject by the similarity matrix at the bottom of Fig 4B.

In contrast to the tongue, we found that only four bases were needed to parsimoniously reconstruct the single trial images of the lips (reconstruction error plateaued at ~ four NMFs). This likely reflects the fact that the tongue is the primary articulator responsible for shaping the vocal tract during vowel production, while the lip is a secondary articulator for vowels (e.g. Fig 3F and 3G). Furthermore, the contribution of these bases to the reconstruction of the different vowels is apparent. For example, basis 1 would contribute to vowels with large lip openings (e.g. /*ɑ*/), while the basis 4 likely contributed to vowels with more narrow lip openings (e.g. /*u*/). However, in general, the lip NMF bases did not have the same qualitative degree of one-to-one correspondence to the mean lip images. Instead, weighted combinations of several lip NMFs would likely have to contribute to the reconstruction of single images. Nonetheless, the different NMF bases for the lips were much less similar to each other than were the mean images for the vowels (Fig 4G and 4H). Across all 6 speakers examined here, similar results for number of bases and similarity of bases were found (S2 Fig).

We examined if the NMF bases are useful for classifying vowels. To this end, we reconstructed each single utterance tongue and lip images as an optimal weighted combination of the NMF bases. This 13-dimensinsional reconstruction weight vector describes the contribution of a given bases to a specific utterance, and can be thought of as the ‘representation’ of that utterance in the NMF bases space. We then took the weight vectors for all utterances within a subject, and used linear discriminants analysis to find the three-dimensional latent space in which the vowels were most linearly separated (as in Fig 3), and projected the data for all vowels into this space. The plot in Fig 4I displays the organization of the vowels in this latent space for the same subject as emphasized in Fig 3. Visual comparison to the plot in Fig 3 suggests that the vowels could be more accurately assigned to distinct classes when using the NMF representation.

Similar results were observed across all subjects. Analogously to the analysis of acoustics and the parametric description of articulator position (Fig 3G), we trained a Naïve Bayes classifier to predict vowel identity based on the projection of the NMF reconstruction weights into the top three latent dimensions from LDA. This was done for the lips and tongue individually, as well as from combined lip and tongue data. The plot in Fig 4J shows the cross-validated classification accuracy of vowels from NMF bases across all subjects. As with the parametric description of the articulators (Fig 3G), the combined model out-performs the individual models (Fig 4J, *: P < 0.05, WSRT, N = 6 for each comparison; median accuracies are 25%, 52%, and 77%). Furthermore, the average classification accuracy utilizing NMF bases was significantly greater than when using pre-defined points (P <0.05, WSRT, N = 6). Therefore, NMF discovers bases that allow for more accurate classification of vowels than using *a priori* defined parametric descriptions of the articulator positions.

### Continuous linear relationship between vowel acoustics and articulator position

**Fig 5:**
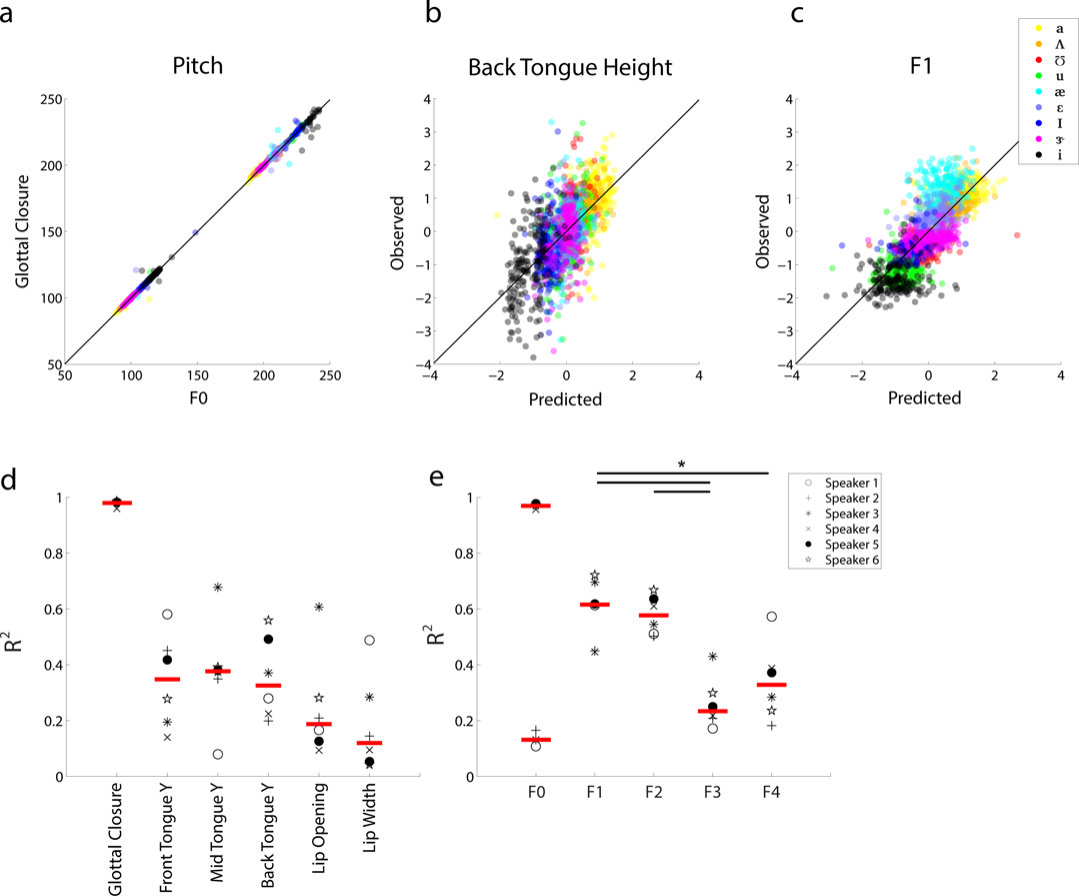
Continuous linear relationship between vowel acoustics and articulator position. **a)** Scatter plot of pitch values (F_0_) vs. frequency of glottal closures from 3 subjects (2 males, 1 female). Color corresponds to vowel identity. **b)** Linear prediction of (z-scored) back tongue height from all acoustic features vs. the observed values for all nine vowels and six speakers. **c)** Linear prediction of (z-scored) F_1_ from all kinematic features vs. the observed values for all nine vowels and six speakers. **d)** Acoustic-to-articulator mappings. Amount of explained variance (R^2^) for six kinematic features from all acoustic features for each subject. Subjects are identified by symbol. **e)** Articulator-to-acoustic mappings. Amount of explained variance (R^2^) for five acoustic features from all kinematic features for each subject.

In addition to the relationship between vowel category and articulatory or acoustic features, we wanted to assess how articulatory features and acoustics continuously map directly to one another. Understanding this relationship is critical because the degeneracies in the transformation between articulatory movements and resulting acoustics are what motivate our methods for explicitly monitoring the articulators. Utilizing regularized linear modeling (see Methods), we evaluated how well a given articulatory feature can be estimated by a linear combination of all acoustic features, and vice versa. An example model prediction is illustrated in Fig 5A–5C. In the most direct relationship, pitch and glottal closure (Fig 5A) are so closely correlated (R^2^ = 0.99) that the link between articulation and acoustics is directly apparent. For the upper vocal tract, back tongue height (Fig 5B) exhibits a more complex relationship with the acoustics, as a linear combination of all acoustic features is capable of modest prediction (R^2^ = 0.36). Similarly F_1_ is fairly well predicted from all articulatory features (Fig 5C, R^2^ = 0.62).

We systematically performed this analysis across all articulatory and acoustic features for all subjects. Performance, as quantified by R^2^, is plotted for each kinematic and acoustic feature, for each speaker, in Fig 5D and 5E (black: individual subjects; red line: median across speakers). The performance of these models illustrates that, on average, front, mid, and back tongue height are best predicted by the acoustics with R^2^ all around 0.40. Lip opening and lip width show more modest values (opening R^2^ = 0.20, width R^2^ = 0.16). For speakers that had glottal recordings collected (N = 3), glottal closure is extremely well predicted by the acoustics (R^2^ = 0.98). F_1_ and F_2_ are best predicted by articulator position (R^2^ = 0.62 and 0.59) and F_3_ and F_4_ show moderate correlations (R^2^ = 0.24 and 0.31). Additionally, F_0_ is predicted very well, but only for those subjects with glottal closure measurement. In general, the features that are well predicted by the linear models are the same features that are the primary contributors to vowel identity (as measured by LDA weights). Importantly, there is significant variability between speakers, suggesting that there is some variability in how each speaker is achieving roughly the same acoustic result. This cross-subject variability has been described before [2,6,7,46], and reiterates the need to explicitly measure articulator movements rather than relying on canonical descriptions of the vocal tract during speech.

### Statistical synthesis of speech from articulator positions

**Fig 6:**
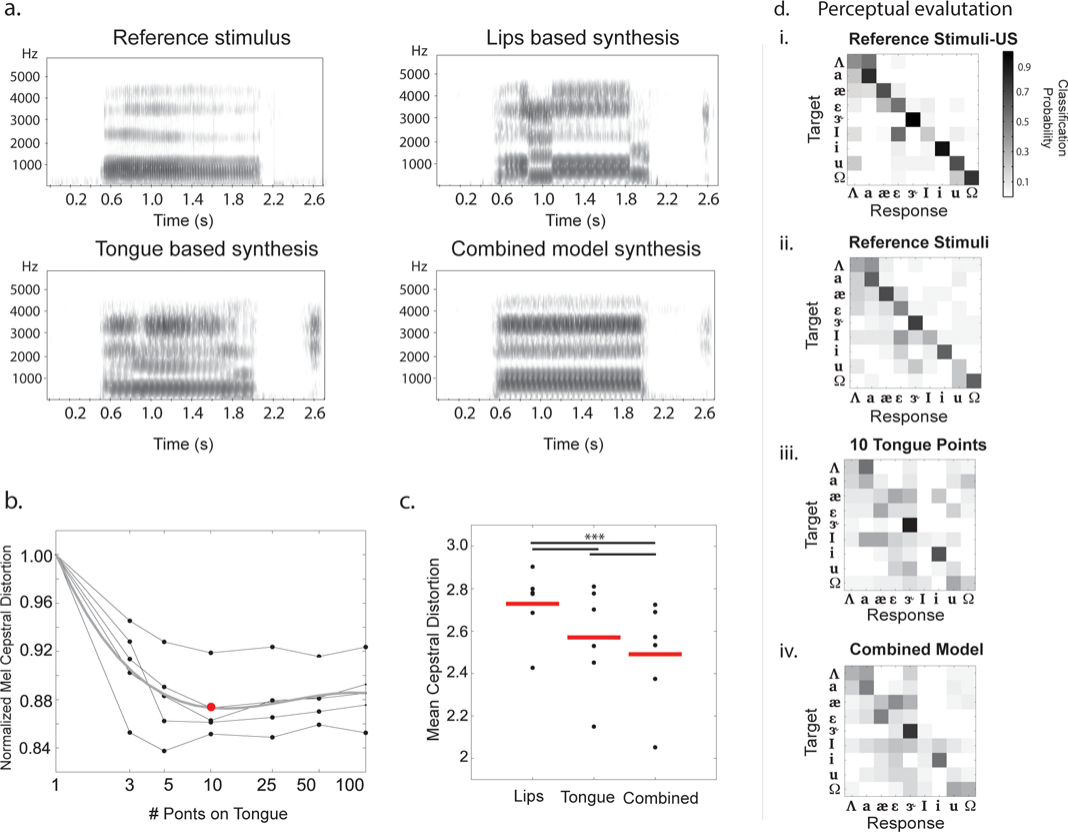
Synthesis of speech from articulator kinematics. **a**) Examples of reference and synthesized stimuli using various articulatory feature sets as identified for articulation of a phoneme /*ɑ*/ by one speaker. **b)** Error on synthesized acoustics, measured as a mean cepstral distortion measure with increasing number of tongue points. Each subject is one black line, overlaid with a grey trend for average across subjects. Red dot at 10 points is selected as the optimal set of tongue points. **c)** Prediction error with different sets of articulators used for synthesis, each subject is one dot and the mean across subjects is marked with the red line segment. **d)** Reference natural stimuli as perceived by listeners based in the United States (i) and Turkers globally (ii) Stimuli synthesized using 10 tongue points (iii) and using both tongue and lip kinematics (iv).

A central long-term goal of our work is to produce a speech prosthetic that transforms recorded brain signals into perceptually meaningful acoustics of speech. As speech production is mediated in the brain through control of the articulators, a first goal is to reconstruct intelligible speech from articulator measurements. This also provides further validation to the usability of the articulator measurements and preprocessing routines described. We use speech synthesis as a tool to evaluate a variety of increasingly rich descriptions of the vocal tract to find the optimal parameter space to generate intelligible and discriminable speech for the vowels considered. Using statistical parametric speech synthesis (see Methods) several articulatory models are evaluated for articulator-to-acoustic conversion. The vocal tract parameterizations considered are i) tongue represented as sequence of equally spaced points (Tongue-based synthesis), ii) features of the lips (Lips-based synthesis), and iii) combined optimal parameterizations of tongue and lip features from (i) and (ii) (Combined model). As a visual illustration of synthesized speech, Fig 6A shows the spectrograms of speech synthesized from each articulatory model considered, shown against a prototypical spectrogram for the reference phoneme /*ɑ*/. It is interesting to note that both the lips and tongue-based models have visible errors in the spectrogram marked by physiologically impossible jumps in spectral energy. However, these errors are compensated in the combined model, giving an insight into the superior performance of the combined model.

To objectively characterize the contributions of individual articulators, cross-validated Mel-Cepstral distortion (MCD) of the predicted speech features on an unseen test set of trials is computed across different models. Fig 6B shows the performance of models solely based on increasing number of points on the tongue. These trends suggest a dense representation using 10 points on the tongue to explain acoustic variability across subjects. Fig 6C compares the individual performances of the optimal tongue and lip based (two mid-sagittal and two coronal extremities of the lips, and derived lip width and lip height as predictors) models. Across subjects, lips and tongue articulators contribute complementary information as shown by superior performance of the combined model. All these comparisons are statistically significant (P < 0.005, WSRT, N = 6).

The ultimate test for speech synthesis is perceptual intelligibility by human listeners. For the case of vowel synthesis here, the right test is a perceptual judgment task to classify each synthesized stimulus into one of the nine possible vowel categories considered. We utilized crowdsourcing to conduct this subjective task (see Methods). 30 samples of unseen trials were synthesized and judged by human listeners on the Amazon Mechanical Turk. Participants were instructed to listen to each sample and identify which of nine vowels they heard. Fig 6D(i) summarizes the results of the perceptual tests as the confusion matrices of the perceived vs. true identities of vowel sounds as reported by listeners in the United States. The same result for listeners not restricted to just the United States is shown in Fig 6D(ii). While it is apparent that prior exposure to the target phonemes, as in the case of American listeners, improves the perceived accuracy, the assessment is still comparable to Fig 6D(i). Even the confusions made are along articulatory lines (e.g. confusions among the low front tongue vowels /*ɛ*/, and /*æ*/; confusions among short and long vowels /*ɪ*/ and /*i*/, /*ʊ*/ and /*u*/ respectively). Hence, subsequent tests are done with no restriction of selecting only American Turkers, since global listeners are still seem to perceive the acoustics (as shown in Fig 6D(ii)) but form a less systematically biased and stricter listener population to assess the identity of these vowels.

We conducted two perceptual experiments on synthetic speech using 10 tongue points (Fig 6D(iii)), and the combined model including 10 tongue points and the lips features (Fig 6D(iv)). The classification accuracies of the synthesized speech are 31% and 36% respectively. It is interesting to note that perceptual classification of natural stimuli is 56% (Fig 6D(ii)) accurate by Turkers around the globe, while the same number restricted to American Listeners is at 64%. It is evident that the overall performance increasingly matches that of natural stimuli with richer descriptions of articulators as anticipated. These also conform to the conclusions of the objective analysis reported in Figs 3G, 4J and 6C; best perceptual identification is obtained with the configuration using the combined tongue and lip features. These perceptual judgment results meet the aforementioned goal of intelligible and discriminable vowels synthesis across speakers, using only their articulatory trajectory information. Thus, from predictions of all articulators, we should be able to synthesize speech.

### Decoding of lip aperture from ECoG recordings during production of words

We have described/validated a system for simultaneous monitoring of all speech articulators, and demonstrated that continuous linear models based on articulator measurements can be used to both predict acoustic features of vowels and to synthesize perceptually identifiable vowel sounds. One of our goals is to use this system to study the neural control of speech articulation by combining the articulatory tracking with simultaneously recorded neural signals from electrocorticography. This is a critical step towards developing a continuously controlled speech prosthetic.

**Fig 7:**
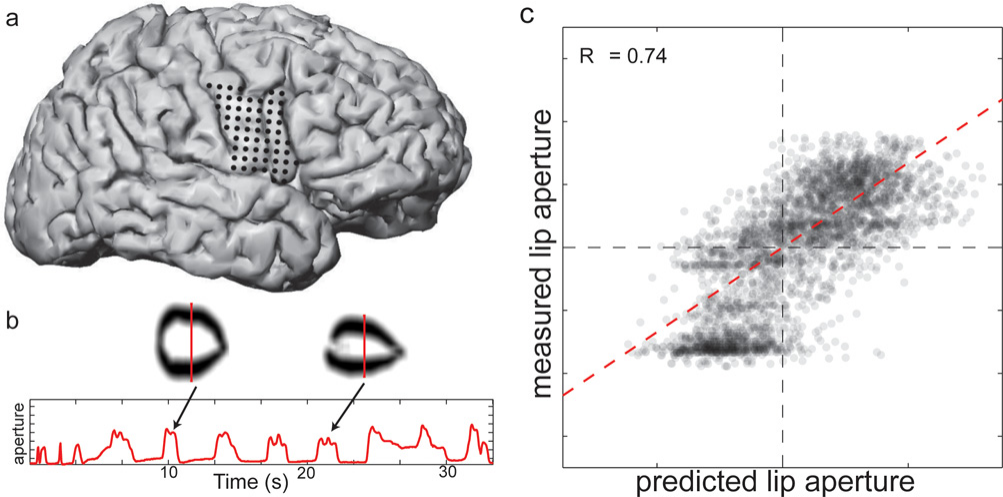
Decoding of lip aperture from ECoG recordings during production of words. **a)** Lateral view of the right hemisphere of a neurosurgical patient. The location of ECoG electrodes over the ventral sensorimotor cortex are demarcated with grey disks. **b)** Example lip shape and vertical aperture during production of words in this subject. **c)** Predicted lip aperture based on linear decoding of ECoG data vs. the actual aperture. Each dot is a time-point; red-dashed line is best linear fit.

To demonstrate the potential of combining articulatory tracking with ECoG recordings, we conducted a preliminary experiment in a neurosurgical patient with our face tracking system. We recorded the cortical field potential from ECoG electrodes placed directly over the ventral sensorimotor cortex (vSMC), an area of the human brain intimately involved in the control of speech articulation and orofacial movements [14,15,19,47,48]. Fig 7A plots a reconstruction of the electrode locations over vSMC in this subject (black dots are electrode locations). At each electrode, we extracted the time-varying high-gamma amplitude (70–150Hz), which likely reflects multi-unit firing rates [49]. We extracted lip aperture from the face tracking system while the patient produced short words. For example, the red trace in Fig 7B plots lip aperture over time during a 35 second segment of the recordings. The lip contours from two vocalizations with different lip apertures are also plotted (Fig 7B, lip aperture is demarcated by the red vertical line in each image). We found that the moment-to-moment aperture of the lips could be well predicted from an optimal linear decoder of the vSMC high-gamma activity (R^2^ = 0.55, Fig 7C). Although it is clear that much more can be done with these recordings, these preliminary results demonstrate the ability to successfully combine our articulator measurement system with ECoG recordings which will allow for studying the neural basis of speech production in unprecedented detail.

## Discussion and Conclusions

We have developed a multi-modal system for simultaneously monitoring the lips, jaw, tongue, and larynx that is compatible with bedside human electrophysiology. To provide initial characterization and validation of our system, we collected and analyzed data from six speakers during the prolonged production vowels. We introduced methods to remove movement artifacts that are a consequence of the recording setting and validated these methods by classifying vowels using canonical descriptions of vowel production. We then applied unsupervised non-negative matrix factorization to derive novel parameterizations of articulator shape and show improved classification accuracy. We complement these categorical analyses by examining the continuous (linear) mappings between acoustics and articulations, and synthesized perceptually identifiable speech acoustics from articulator measurements. Finally, we demonstrate the ability of this system to be used in conjunction with ECoG recordings by robustly decoding measured articulator kinematics from neural activity. Not only will this system allow for unprecedented insight into the neural basis of speech motor control, but also the methods outlined here are relevant for articulator monitoring in any setting where bulky and constrictive equipment is impractical.

### System and methods for data collection and registration

Exploring the relationship between neural activity and measured speech articulator movements is critical to understanding speech motor control. The inability to unambiguously infer movements from acoustics means that we need to explicitly monitor the speech articulators simultaneously with neural activity and acoustics if we are to achieve a complete neurobiological understanding of speech motor control.

A variety of systems have been developed for the purpose of monitoring the speech articulators, but most are impractical for use in conjunction with bedside human electrophysiological recordings; they are either too invasive (e.g. photoglottography [50]), not portable (e.g. real-time MRI [51]), likely to create electrical artifact on the neural recordings (e.g electromagnetic midsagittal articulography [52], or some combination thereof. To address this, we developed a system to simultaneously monitor the lips and jaw with a video camera, the tongue with ultrasound, and the larynx with electroglottography. These methods provide high spatial and temporal resolution, and are fully compatible with beside ECoG recordings in a hospital setting. Another important consideration when recording in the clinic is that data can only be collected in short sessions, and it is not possible to ensure that the recording apparatus is positioned in the same way relative to the speaker in each session, or even across vocalizations within a session. We note that this is a common issue for all those who work with multi-channel, multi-site recordings of behavior. To address this general issue, we have developed two algorithms to register data across sessions and conditions. A critical assumption of our registration methods is that the position of the articulators several hundred milliseconds before acoustic onset can be used as a neutral reference for subsequent time points. Another assumption is that the registration transformations are restricted to scaling’s, translations and rotations (i.e. an affine transform).

We thoroughly validated this system and methods of registration by employing them on six subjects speaking nine vowels. We show that our system measures articulator movements that are consistent with previous studies of vowel production. First, the vowels organize according to kinematic and acoustic features as previously described (e.g. /i/ is characterized as a ‘high front vowel’). Second, when relating articulator kinematics to produced acoustics, the classic features of tongue height and frontness prove to be the best predictors of vowel acoustics. Lastly, we consistently see variability between individual speakers. Together, these results validate our system and reiterate the need to explicitly measure articulation rather than relying on categorical descriptions of speech production.

It should be noted that our system does have some limitations. First, we are not able to image the entirety of the vocal tract, and therefore unable to describe the vocal tract as completely as some techniques allow [53]. Furthermore we are imaging a two-dimensional sagittal plane of the tongue, and while this plane has been shown to be a good descriptor of tongue kinematics, it is known that the non-sagittal shape of the tongue has an important impact on the resulting acoustics [54]. Lastly, the placement of the ultrasound transducer under the speaker’s jaw slightly restricts natural jaw motion, which likely results in some degree of compensatory movement from the other articulators. These limitations are unavoidable given the clinical constraints of the recording setting, and are shared with most other dynamic articulator monitoring techniques. In this study we have chosen to primarily focus on the vowels for validation purposes. The articulations involved in vowel production correspond to a reduced sub-space of the total articulatory space of speech. Our methods should be extendable to the analysis to the entire English inventory, which is an important future direction. Furthermore, it will be important to derive time varying relationships between articulators and acoustics [55–57]. This could be done using a combination of autoregressive models and canonical correlation analysis.

### Non-negative matrix factorization extraction of vocal tract bases

The generation of even the simplest speech sounds requires the precise coordination of multiple articulators to achieve a vocal tract shape that produces the target sound. Indeed, it has been argued that speech motor control should be viewed in terms of dynamic spatial configurations of articulators [15,58]. Non-negative matrix factorization (NMF) has been used to extract ‘meaningful’ bases from data that consist of only positive values, such as images and movies, or recordings of electromyographic recordings [34,59]. Recently, a variant of NMF (sparse convolutional NMF) was used to model real-time MRI data of human speech production for extraction of time-varying spatial configurations of the vocal tract [60]. Here, we applied NMF to the lip and tongue data during the center of the vowel, and found that the extracted bases had several desirable qualities: 1) several bases resembled the mean shapes associated with specific vowels (Fig 4), 2) the individual bases were less similar to each other than the means shapes of individual vowels (Fig 4), and 3) NMF bases could be used to improve classification performance over *a priori* defined point-based descriptions (Fig 4). Indeed, using the NMF bases, the accuracy for classification of vowel identity approached the accuracy based on the acoustics. Together, these results demonstrate the utility of NMF for data-driven extraction of vocal tract bases, and suggest it would be a useful approach for understanding other types of behavioral data.

Our utilization of NMF to extract purely spatial bases was motivated both to parallel the analysis of kinematic/acoustic features during the center of the vowel, but also to provide clear demonstration of the utility of this method to extract interpretable bases that reflect important vocal tract shapes. In this work, we extracted bases for the lips and tongue separately, and found that this resulted in readily identifiable bases for each articulator. We derived separate bases for lips and tongue because NMF attempts to explicitly reconstruct every point in the data, and the number of data points associated with lips was much larger than the number of data points associated with the tongue. Therefore, in a combined analysis, NMF would have ‘weighted’ the lips more heavily than the tongue, making it difficult to interpret the bases. Generally speaking, consideration of how the objective function of an algorithm interacts with the statistics of the data is critical for interpreting its outputs. Nonetheless, in combination with previous studies, our results strongly suggest that NMF will be a fruitful analytic approach for understanding speech production [60]. A critically important direction of future research is to use data driven descriptions of the vocal tract to understand the cortical control of speech through direct encoding/decoding analysis of simultaneously collected neural activity from multiple subjects.

### Statistical speech synthesis from articulators to acoustics

The speech synthesis experiments reported in this work show that the processed articulatory trajectories retain sufficient information to synthesize audio that can be perceived as the intended vowel. Also noteworthy is the result that not all points on the tongue are necessary to accomplish this goal, suggesting that a high spatial resolution of tongue need not be tracked or estimated for intelligible synthesis. These findings are also shown to be consistent across subjects setting a valid precedent for synthesis of all possible phonemes using only articulatory data. At the implementation level, the advantage of the statistical model used for speech synthesis is that it is not constrained to a predefined geometrical or physiological model of the vocal tract (which may not be tractable), but instead models the salient relationships between articulatory and acoustic feature streams, as inferred from the data. Another advantage is that statistical models can be bootstrapped and adapted across speakers, potentially reducing the amount of data required to train the synthesizers [61]. It remains to be shown that this success can also be replicated on synthesizing consonants, where place of constriction (e.g. velar, palatal etc.) and the manner (affricate, plosive etc.) play additional roles along with the overall shape of the tongue and lips used here. Nonetheless, our results imply that decoding trajectories of the critical articulators from neural activity can be sufficient to produce intelligible speech.

### Decoding of speech kinematics

Here, we provide the first direct demonstration that the moment-to-moment kinematics of a vocal tract feature can be well predicted from a statistical mapping of the vSMC high-gamma activity (Fig 7). Brain-machine interface approaches to speech prosthetics hold promise to dramatically improve the communication abilities of the profoundly disabled [62]. Together with previous studies, our results strongly suggest that combining continuous statistical mappings of vSMC activity to articulator kinematics with mappings of kinematics to acoustics is likely to be a successful strategy for a brain-machine interface for a speech prosthetic. A continuous transformation approach could be combined with a categorical decoder for a hybrid prosthetic system. The advantages of such a hybrid system would be the capacity to simultaneously model the continuous transformation of vSMC activity into speech (which is the ‘natural’ transformation), while capitalizing upon existing massive data sets to incorporate a language model for classification (as has been powerfully applied to automated speech recognition systems). Future studies combining ECoG recordings with simultaneous measurement of multiple vocal tract articulators, as permitted by our system, would allow unraveling the cortical coordination underlying multi-articulator control for speech production. Understanding whether neural representations in different brain areas underlying speech production are acoustic, articulatory, or phonetic is a central challenge in developing a cortical theory of speech production. The ability to measure speech behavior on the acoustic, articulatory and phonetic basis with the simultaneously collected high spatio-temporal resolution cortical activity on an individual subject and single-utterance level may provide insight into this issue.

## Supporting Information

S1 Fig LDA Loading Values. Loading magnitudes resulting from LDA performed on the middle fifth of the vowel for formant (a) and articulatory (b) features, for the first 3 latent dimensions. LDs are ordered according to their ability to discriminate between vowels. Black line denotes the median value across speakers. Horizontal lines with asterisks denote features with significant differences in loading magnitude distributions (P < 0.05, WSRT, N = 6).
(TIF)

S2 Fig Number of NMF Bases. Similar results were generally found across all subjects. In S2a and b Figs, we plot the cross-validated reconstruction error of tongue and lip images, respectively, as a function of the number of NMF bases used in the reconstruction for each speaker (black lines, mean ± s.d. from bootstrap), as well as the best fitting exponentially decaying function (grey line). Across subjects, we found that reconstruction error reached approximate asymptote (red squares) after nine NMFs for the tongue and four NMFs for the lips, indicating that nine and four NMFs was a parsimonious number of bases to use. In c, we plot the distributions of shape similarity for mean and NMF tongues and lips. Distributions show: median (red), 25/75 percentiles (black box), 95% CI, dashed lines, outliers (grey ‘+’). NMFs provide a more dissimilar description of the vocal tract shapes.
(TIF)
