# Supplementary figures and images for: High-Resolution, Non-Invasive Imaging of Upper Vocal Tract Articulators Compatible with Human Brain Recordings
Source: PLoS One. 2016 Mar 28;11(3):e0151327. doi: 10.1371/journal.pone.0151327 (PMC4809489; doi:10.1371/journal.pone.0151327)

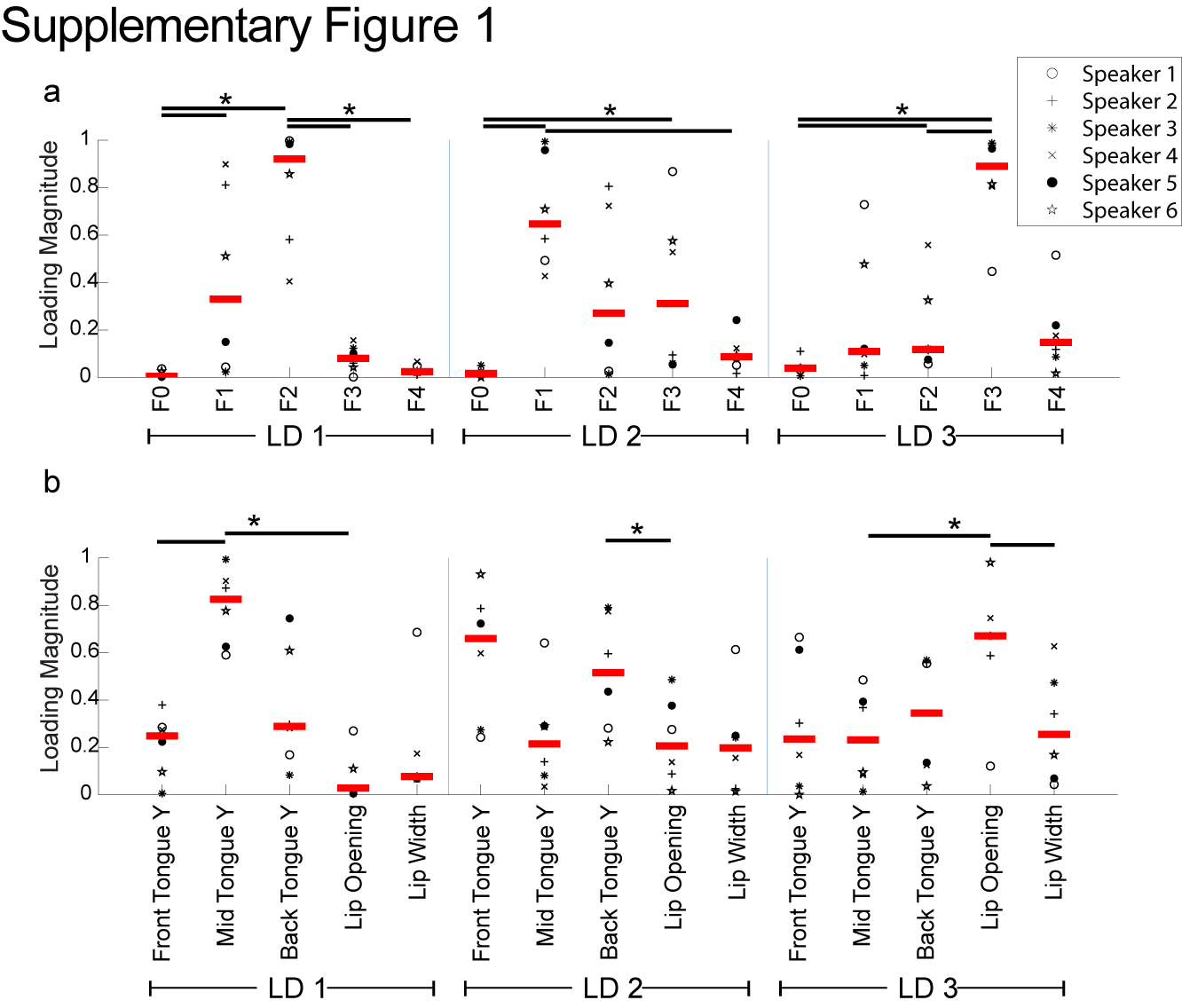

Supplement: S1 Fig — Loading magnitudes resulting from LDA performed on the middle fifth of the vowel for formant (a) and articulatory (b) features, for the first 3 latent dimensions. LDs are ordered according to their ability to discriminate between vowels. Black line denotes the median value across speakers. Horizontal lines with asterisks denote features with significant differences in loading magnitude distributions (P < 0.05, WSRT, N = 6). (TIF) [file pone.0151327.s001.tif]

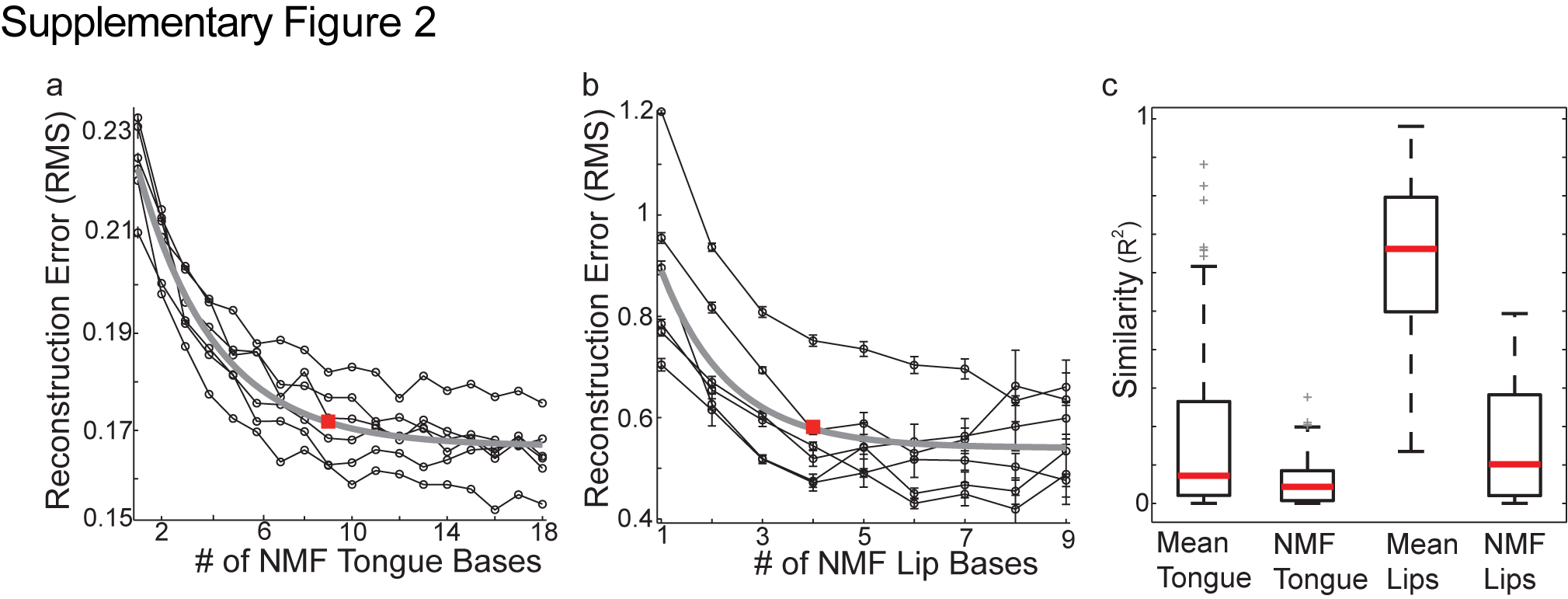

Supplement: S2 Fig — Similar results were generally found across all subjects. In S2a and b Figs, we plot the cross-validated reconstruction error of tongue and lip images, respectively, as a function of the number of NMF bases used in the reconstruction for each speaker (black lines, mean ± s.d. from bootstrap), as well as the best fitting exponentially decaying function (grey line). Across subjects, we found that reconstruction error reached approximate asymptote (red squares) after nine NMFs for the tongue and four NMFs for the lips, indicating that nine and four NMFs was a parsimonious number of bases to use. In c, we plot the distributions of shape similarity for mean and NMF tongues and lips. Distributions show: median (red), 25/75 percentiles (black box), 95% CI, dashed lines, outliers (grey ‘+’). NMFs provide a more dissimilar description of the vocal tract shapes. (TIF) [file pone.0151327.s002.tif]
